# The interplay between mineral metabolism, vascular calcification and inflammation in Chronic Kidney Disease (CKD): challenging old concepts with new facts

**DOI:** 10.18632/aging.102046

**Published:** 2019-06-26

**Authors:** Carla Viegas, Nuna Araújo, Catarina Marreiros, Dina Simes

**Affiliations:** 1 Centre of Marine Sciences (CCMAR), University of Algarve, Faro 8005-139, Portugal; 2 GenoGla Diagnostics, Centre of Marine Sciences (CCMAR), University of Algarve, Faro 8005-139, Portugal

**Keywords:** chronic kidney disease, cardiovascular disease, vascular calcification and inflammation, vitamin K-dependent proteins, calciprotein particles, extracellular vesicles

## Abstract

Chronic kidney disease (CKD) is one of the most powerful predictors of premature cardiovascular disease (CVD), with heightened susceptibility to vascular intimal and medial calcification associated with a high cardiovascular mortality. Abnormal mineral metabolism of calcium (Ca) and phosphate (P) and underlying (dys)regulated hormonal control in CKD-mineral and bone disorder (MBD) is often accompanied by bone loss and increased vascular calcification (VC). While VC is known to be a multifactorial process and a major risk factor for CVD, the view of primary triggers and molecular mechanisms complexity has been shifting with novel scientific knowledge over the last years. In this review we highlight the importance of calcium-phosphate (CaP) mineral crystals in VC with an integrated view over the complexity of CKD, while discuss past and recent literature aiming to highlight novel horizons on this major health burden. Exacerbated VC in CKD patients might result from several interconnected mechanisms involving abnormal mineral metabolism, dysregulation of endogenous calcification inhibitors and inflammatory pathways, which function in a feedback loop driving disease progression and cardiovascular outcomes. We propose that novel approaches targeting simultaneously VC and inflammation might represent valuable new prognostic tools and targets for therapeutics and management of cardiovascular risk in the CKD population.

## Introduction

Cardiovascular disease (CVD) and chronic kidney disease (CKD), also known as chronic or non-communicable diseases, are leading causes of disability and death all over the world. While CVD is the major cause of morbidity and mortality in the developing world, representing a tremendous social and economic burden, it is also the most common death cause in the CKD population. CKD is an important public health problem that is characterized by poor health outcomes and very high health care costs. Since the prevalence of CKD and CVD is higher in older people and life expectation is increasing, managing effective diagnosis and treatment for these highly prevalent diseases is crucial to impact the health of aging population.

CKD is defined as a group of abnormalities that can affect the kidneys structure or function and are present for more than 3 months with health implications [1]. CKD affects approximately 14% of the adult population in the United States, where it aligns closely with the prevalence of diabetes and hypertension [2]. Details from the ERA-EDTA registry annual report for 2015 show that 70 million Europeans have partially lost their kidney function and are at high risk of becoming dependent on renal replacement therapies (dialysis or transplantation) [3]. Additionally, Global Burden of Disease 2015 study estimated that 5–10 million people die annually from kidney disease and high-income countries typically spend more than 2–3% of their annual health care budget on the treatment of end-stage kidney disease [4]. Low kidney function is linked to poor health outcomes, with clinical manifestations in a wide variety of other organ systems, including endocrine, nervous, gastrointestinal, musculoskeletal, and hematologic, and associated to a much higher risk of cardiovascular disease. The risk of cardiovascular disease exponentially increases as kidney function declines, being the major contributor to the high incidence of cardiovascular complications and death in this population [5–7]. This is partially due to vascular calcification (VC) and accelerated atherosclerosis, as a result of the mineral and bone disorder (MBD) that often accompanies low kidney function and complicates CKD. CKD-MBD involves changes in mineral ion homeostasis, bone quality and turnover, and extraskeletal calcification [8,9].

Epidemiologically, CKD, diabetes mellitus, and atherosclerosis are the clinical conditions that most contribute towards development of VC of medial and intimal layers of the vessel wall. In patients with CKD, VC is associated with significant morbidity and mortality and is one of the strongest predictors of cardiovascular risk [10,11]. The prevalence of VC increases as glomerular filtration rate (GFR) declines and calcification processes occur years earlier in CKD patients than in the general population [12]. The impact of VC on cardiovascular outcome relates to the location of mineral deposition. Intimal calcification reflects atherosclerotic plaque burden and may influence plaque rupture, being a strong predictor of cardiovascular events and mortality. On the other hand, medial calcification induces stiffening of the vessel, increased pulse pressure, left ventricular hypertrophy, and can result in heart failure. In dialysis patients, medial calcification is closely associated with the duration of hemodialysis and calcium–phosphate disorders [13]. Both forms of calcification are prominent and can occur simultaneously, contributing to the increased cardiovascular mortality in CKD [14]. In addition, the risk associated with progression and severity of atheromatous plaques has been shown to be prevalent in CKD patients, considered to develop accelerated atherosclerosis [15].

Although many aspects concerning the pathogenesis of VC are still unclear, it is currently accepted that it is an active and multifactorial process, which must be highly controlled and constantly inhibited. Several pathological features are widely described to be associated with VC. The proliferation, differentiation and apoptosis of vascular smooth muscle cells (VSMCs), oxidative stress, endothelial dysfunction, increased extracellular matrix (ECM) remodeling, release of calcifying extracellular vesicles (EVs), loss of mineralization inhibitors, and chronic inflammation, are well known contributors to its development [16]. In CKD patients, VC is exacerbated as a result of several interconnected mechanisms involving abnormal mineral metabolism, dysregulation of endogenous calcification inhibitors and inflammatory pathways.

In this review we will integrate the knowledge on the formation of calcium-phosphate (CaP) mineral crystals with dysregulated mineral metabolism, the importance of mineralization inhibitors on mineral formation and maturation, and their effect on VC and inflammation, associated with cardiovascular outcomes in CKD. Simultaneously, we discuss the immediate and long-term effects of widely used therapeutic strategies associated with the control of dysregulated mineral metabolism, such as dietary restrictions and phosphate binders (PBs), in light of their effect on VC. Clearly, VC and inflammation not only play a key role in CKD pathophysiology and cardiovascular disease but are also involved in a complex bidirectional crosstalk ultimately leading to disease progression. A deeper knowledge on the molecular mechanisms and the discovery of new modulating agents targeting both inflammation and calcification, will pave the way to the discovery of new biomarkers and therapeutic strategies for cardiovascular-associated diseases.

## The importance of calcium (Ca) and phosphate (P) minerals in CKD: balance and regulation

P and Ca are absolutely essential for life but its balance and regulation are tightly controlled because both positive and negative balances have detrimental clinical implications. In patients with CKD, a negative balance of Ca and P favors bone mineral loss and osteoporosis, increased risk for bone fragility and fractures. On the other hand, a positive balance favors soft tissue calcification, consequent cardiovascular events, and interrelates with high morbidity and mortality.

### Ca and P homeostasis

Ca and P are absorbed from the diet in the intestine, stored in the skeleton, and reabsorbed and excreted by the kidneys (extensively reviewed elsewhere [17–19]). In healthy adults, normal daily intake of Ca and P is considered in the range of 800–1000 mg and 700-2000 mg, respectively. The majority of Ca and P stores in the body are in the bone (approximately 99% and 85%, respectively), while the remaining is present in the extracellular and intracellular spaces, including the circulating fractions (approximately 0.1%). In adulthood, normal total serum Ca is approximately 8.5–10 mg/dL and includes the ionized and complexed fractions, as well as the protein-bound fraction. Normal serum P levels are considered between 2.5–4.5 mg/dL. Among the many factors that can influence Ca and P homeostasis, high dietary Ca and P intake are related with increased serum Ca and P, which are associated with poor clinical outcomes including increased CVD risk [20–23].

In normal physiology, Ca and P homeostasis are regulated through hormonal control of a four-tissue axis involving intestine, bone, kidney, and parathyroid gland, that tightly control serum ionized Ca and P levels within a narrow range.

**Figure 1:**
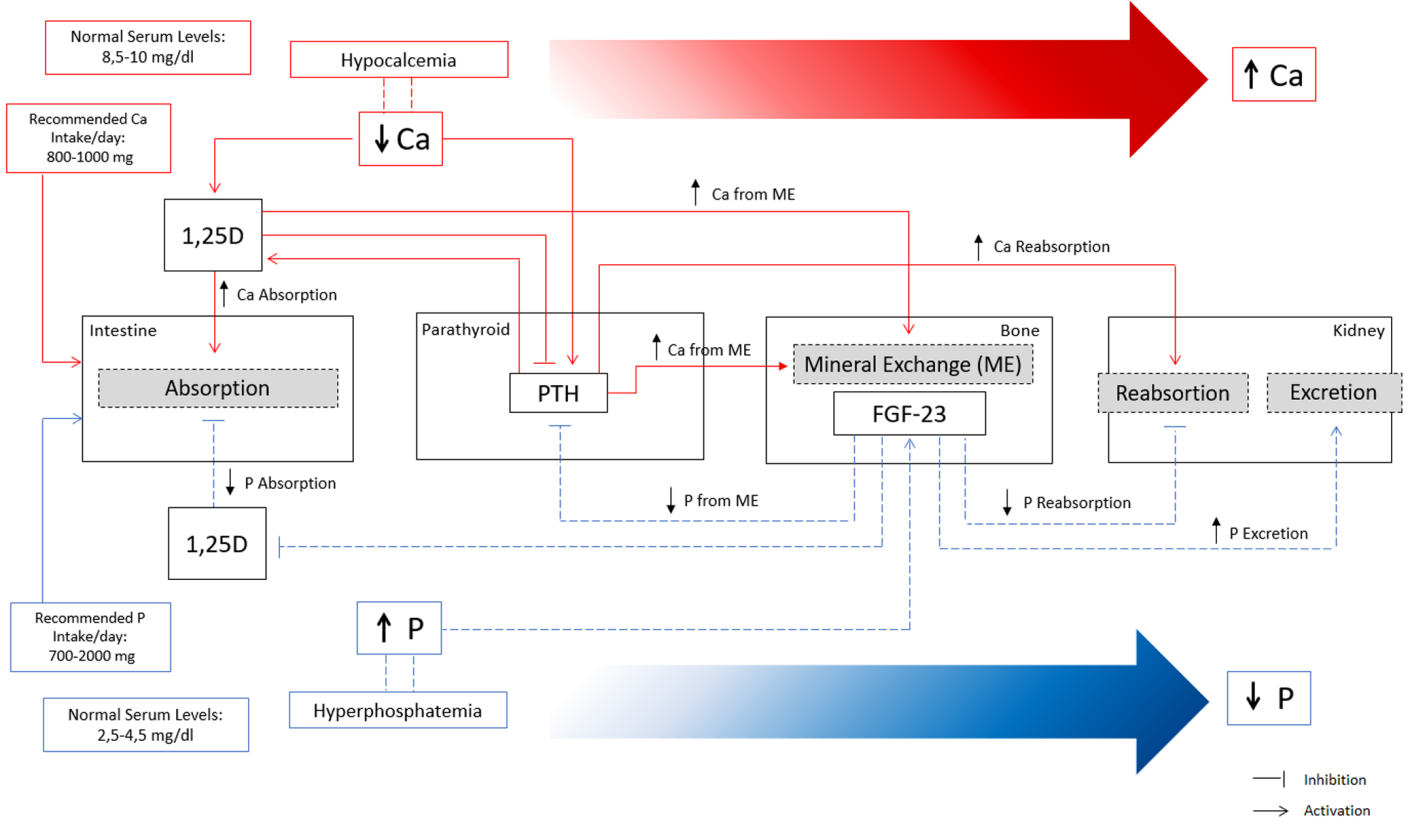
**Overview of calcium (Ca) and phosphate (P) homeostatic regulation.** Red lines represent the main mechanisms of Ca regulation in a situation of hypocalcemia. Decreased Ca levels in serum increase 1,25D and PTH. Increased levels of 1,25D increase Ca absorption at the intestine and stimulate mineral exchange in bone increasing Ca efflux. Increased PTH stimulate mineral exchange in bone increasing Ca efflux, and increase Ca reabsorption in the kidney. Indirectly, high levels of PTH stimulate 1,25D with consequent increase in Ca absorption. Overall, the concerted action of PTH and 1,25D lead to increased serum Ca levels until the normal range by increasing Ca reabsorption at the intestine, increasing Ca from mineral exchange in bone, and increasing Ca reabsorption in the kidneys. Blue lines represent the main mechanisms of P regulation in a situation of hyperphosphatemia. High levels of serum P increase FGF-23 production in bone, which exerts several effects to promote a decrease in serum P. FGF-23 decreases P reabsorption and increases P excretion in the kidneys, and indirectly decreases P absorption at the intestine and P efflux from bone mineral exchange, through the inhibition of 1,25D and PTH.

The two primary hormones involved in calcium balance are parathyroid hormone (PTH) and 1,25 vitamin D (1,25D) (Figure 1). PTH secretion is stimulated by hypocalcemia and suppressed by hypercalcemia. Low levels of serum ionized Ca are sensed by the calcium-sensing receptors (CaSR) on the parathyroid gland, which stimulates PTH synthesis and secretion. In order to raise serum Ca to normal levels, PTH exerts effects at multiple levels such as: (i) stimulating bone osteoclast activity to increase Ca efflux from bone; (ii) increasing Ca reabsorption in the kidney; (iii) stimulating 1,25D production, which in turns increases intestinal Ca absorption and Ca efflux from bone. Hypercalcemia suppresses PTH secretion and stimulates CaSR activity to enhance renal Ca excretion.

The primary regulator of P homeostasis is the kidney which adjusts the excretion to the dietary load of P. The main hormones implicated in regulation of serum P are fibroblast growth factor 23 (FGF-23) with its cofactor Klotho, PTH, and 1,25D (Figure 1). FGF-23 is primarily a phosphaturic hormone, produced by osteoblasts and osteocytes under physiological conditions, and secreted from bone in response to increased serum P and 1,25D. FGF23 exerts several effects to promote a decrease in serum P such as (i) inhibiting P reabsorption in the kidney by suppression of type II sodium phosphate cotransporters; (ii) decreasing P absorption by inhibiting 1,25D production; (iii) suppressing PTH and expression of the CaSR and vitamin-D receptor in the parathyroid gland. In addition, FGF-23 increases Ca reabsorption in the kidneys, probably to compensate for low Ca absorption due to induced 1,25D deficiency. PTH increases phosphate excretion by stimulating FGF-23 production and reducing type II sodium phosphate cotransporters.

Special attention and concerns have been raised in recent years in relation to the possible excess of P intake in the general population, and its correlation with disturbances in bone and mineral metabolism, compromising bone health [24]. In addition to a high protein diet, the increased consumption of processed food and the use of food additives containing high levels of inorganic phosphates, have been linked to acute spikes or transient increases in serum P. This might be partially explained by the high rate of absorption efficiency, particularly of inorganic P present in food additives, and the circadian fluctuation in serum P. In fact, short term high P diets have been shown to significantly increase circulating levels of FGF-23 and bone related markers in human and mice, while decrease bone mineral density in mice [25]. Increased FGF-23 levels in individuals with normal renal function have been related with renal P wasting and impaired bone mineralization [26]. Excessive intake of P has been linked to increased levels of serum P, which in turn is suggested to be the main stimulus for phosphorus homeostasis disruption. Consequently, in both healthy and renal disease individuals (detailed below), current dietary habits might contribute to current worldwide burdens of osteoporosis and renal dysfunction.

### Dysregulation of mineral metabolism in CKD: the mineralization paradox behind CKD-MBD

In the CKD context, the ability to filter and excrete P is progressively compromised as kidney function declines, leading to a progressive dysregulation of the intricate set of feedback loops that tightly regulate Ca and P homeostasis. Hyperphosphatemia is usually defined as serum P >=5.0 mg/dL, and hypercalcemia as serum Ca >=10 mg/dL [27,28]. However, P accumulation has been suggested to occur since early stages of CKD, prior to the development of hyperphosphatemia. This situation is prevented by the adaptive and compensatory mechanisms that increase P excretion through the increased phosphaturic action mediated by FGF-23/klotho axis and PTH. In fact, increased serum P, although still within the normal range, has been correlated with adverse cardiovascular and renal outcomes and overall survival in early stage CKD patients [29–34]. In stages 2–5 CKD patients, the reported thresholds of serum P shown to predict adverse outcomes ranged between 3.5 and 4.6mg/dL. FGF-23 levels increase since stage 2 and continue to rise as CKD progresses. In addition, a gradual increase in serum P can be observed since the beginning of stage 3. When GFR is below 30 mL/min/1.73 m2, the adaptive mechanisms are unable to sustain serum P in the normal range due to an imbalance between P intake and renal excretion capacity. As kidney function declines, FGF-23 and then PTH levels increases in CKD, exerting opposing effects on vitamin D metabolism. Although PTH stimulates 1,25D production, increasing FGF-23 creates a state of 1,25D deficiency that decreases serum Ca and further increases PTH. In CKD stage 5, FGF-23 levels are normally several hundred folds above the normal range [30,35]. Since hypocalcemia stimulates PTH production and decreased levels of 1,25D results in failure to inhibit PTH synthesis, an excessive rise in PTH release often accompanied by parathyroid hyperplasia, is common in CKD patients and referred as secondary hyperparathyroidism (SHPT) [36]. As kidney function declines, phosphate retention occurs, and hyperphosphatemia has been consistently associated with CKD progression and cardiovascular outcomes. However, a state of hypocalcemia is often associated with these patients, despite it is quite uncommon in CKD stage 3 and early stage 4, and more often observed in stage 5 [34,37]. One important issue is that the levels of ionized serum Ca might not reflect Ca balance in the organism [19]. Since only 0.1% of total body Ca is present in circulation, the determination of Ca balance or total body Ca content, or adequacy of Ca load, cannot be infer by serum Ca levels. Indeed, the constant stimulation of Ca and P efflux from bone, the increased Ca reabsorption and the decreased urinary Ca excretion capacity might originate a positive calcium balance. At physiological pH, free Ca is able to bind to excessive P and facilitate P removal, although the resulting calcium-phosphate mineral (CaP) does not precipitate directly from solution into hydroxyapatite that can be deposited within soft tissues. Instead, it passes through a series of phases including the transition from an initial unstable amorphous calcium phosphate (ACP-1) into a more stable and less soluble form of amorphous calcium phosphate phase (ACP-2).

**Figure 2:**
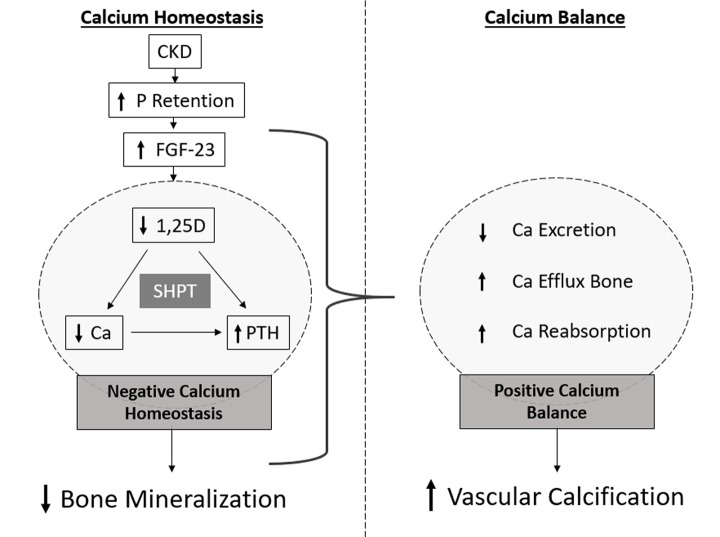
**Schematic representation of the mineralization paradox in CKD.** As kidney functional declines, P retention occurs and FGF-23 highly increases, with consequent decrease in 1,25D levels. Dysregulated levels of 1,25D lead to increased levels of PTH and decreased levels of serum Ca. Low levels of serum Ca maintained by low 1,25D, constantly stimulate PTH production, often resulting in secondary hyperparathyroidism (SHPT) and bone resorption leading to decreased bone mineralization. However, increased Ca efflux from bone, and increased Ca reabsorption and decreased excretion in the kidneys originate a positive Ca balance, correlated with increased vascular calcification.

Of note, the concept of calcium-phosphate product, often referred as CaxP, widely used in the literature, is obtained by simply multiplying serum Ca and P levels and does not entirely reflect the aggregation of Ca and P in soft tissues, here referred as CaP. This emphasizes the significant difference between Ca homeostasis and balance. While compensatory mechanisms are crucial to counteract hyperphosphatemia in short-term, these adaptive responses may increase the risk of undesirable and detrimental effects resulting from a positive calcium balance. Ultimately, this will lead to a process of soft tissue calcification, increasing the cardiovascular risk. These adaptive mechanisms are effective in maintaining serum P and Ca within the normal range until late stages of CKD. This situation constitutes a major risk for CKD patients since the accumulation of P, Ca and CaP progresses since early stages of disease development. Often, at the time of diagnosis, bone loss might be significant and soft tissue calcification already established. This process is often referred as the mineralization paradox in CKD (Figure 2). Clinical studies have shown that the development of VC is correlated with a decrease in bone mineral density, the incidence of bone fractures and mortality in CKD patients [38,39]. Current Kidney Disease Improving Global Outcomes guidelines defined that CKD-MBD should be used to describe the broader clinical syndrome encompassing mineral, bone, and calcific cardiovascular abnormalities that develop as a complication of CKD [8].

## Controlling hyperphosphatemia in CKD patients: the true challenge in vascular calcification management?

Large epidemiological trials support the fact that elevated serum P concentrations are associated with all-cause and cardiovascular mortality, especially in patients on dialysis, but also in predialysis patients and even in the normal population. Higher serum P is associated with more extensive coronary artery calcium and coronary occlusion in CKD patients, death and CVD events in CKD patients and in the general population [30,40–42]. In this context is conceivable to assume that controlling serum P levels may translate in less calcification and adverse CVD outcomes, and this has been the therapeutic strategy hallmark in CKD patients over the years. Therapeutic measures to control serum P levels in CKD are mainly focused on dietary restrictions to decrease P load, and supplementation with phosphate binders to reduce intestinal P absorption.

### Collateral effect of dietary restrictions in CKD patients: vitamin K deficiency

CKD progression towards a chronic renal insufficiency has been therapeutically approached by dietary interventions, aiming to decrease the loading of P, sodium, potassium and reduce uremic toxin retention. Dietary restrictions are often adapted according to CKD developmental stages. Healthy dietary approaches, like Dietary Approaches to Stop Hypertension and the Mediterranean protective diet, rich in antioxidant species, may be beneficial for CKD prevention and early disease stages [43]. With CKD progression, the regulation of protein intake becomes more restricted and often includes the use of low to very low-protein regimes. In that case, supplementation with essential amino acids and keto-acids is important to assure an adequate essential amino acid supply [44,45]. In end-stage renal disease nearly all patients are prescribed with dietary P restrictions to control hyperphosphatemia [46]. Current Kidney Disease Improving Global Outcomes guidelines recommend limiting dietary P intake as a first-line therapy for treatment of hyperphosphatemia and secondary hyperparathyroidism [14]. Although these dietary approaches are set to control protein, sodium, potassium and P intake, several studies correlate CKD-related pathologies such as MBD, cardiovascular diseases, anemia and inflammation, with a deficit of micronutrient contents associated with these recommended diets and contributing to morbidity and mortality in this population [47,48]. In fact, while prescribed P dietary restrictions are often associated with decreased serum P levels, the overall improved survival among hemodialysis patients has been questioned, and important concerns related with its association with increased mortality, have been raised [46]. To achieve the lower dietary load of potassium and P, CKD dietary plans are poor in vegetables, fruit, milk derivatives, yogurt, cheese, limiting the source of several micronutrients relevant for cardiovascular health, such as vitamin K. Vitamin K is a family of fat-soluble molecules consisting on the natural available forms phylloquinone (vitamin K1) and menaquinones (vitamin K2) and the synthetic form, menadione (vitamin K3) [49].

Vitamin K1 is found mainly in green leafy vegetables, but also in some fruits, like avocado, kiwi and grape, as well as in olive and soybean oils. Vitamin K2 is mainly synthesized by intestinal bacteria (with the exception of menaquinone 4 (MK-4) that is directly converted from vitamin K1 [50]), and it can be mostly found in fermented soy beans (well known as natto), followed by dairy products. Other sources of vitamin K2 are chicken meat, egg yolks, beef and salmon. In the western diet, vitamin K1 is about ten times more available then vitamin K2, although only 10% of the ingested vitamin K1 is absorbed [51,52]. This data is not available for vitamin K2 not only because K1 predominates in diet, but also because K1 is the form routinely measured in blood [53]. Although menaquinones in plasma are mostly undetectable, MK-7 is often detected in serum of people eating natto in a regular base, which could be explained by its longer plasma half-life [54]. Vitamin K status is dependent on the dietetic intake, but also on intestinal bacterial synthesis, highlighting the importance of the CKD dietary plan in the intestinal microbiota health. The recommended adequate intake of vitamin K is restricted to vitamin K1 quantification, and has been proposed as 1 µg of phylloquinone/kg body weight per day, according to the Panel on Dietetic Products, Nutrition and Allergies of the European Food Safety Authority and The Institute of Medicine of the United States [55].

Vitamin K is an essential cofactor for the post-translational modification of vitamin K-dependent proteins (VKDPs), where specific glutamic acid (Glu) residues are modified to γ-carboxyglutamic acid (Gla) residues, by the γ-glutamyl carboxylase enzyme [49]. Insufficient γ-carboxylation of VKDPs with a known role as regulators of soft tissue mineralization, has been widely linked to increased VC and cardiovascular diseases [56–58]. In fact, vitamin K status has been evaluated through the quantification of circulating vitamin K1 and K2, and/or indirectly through the quantification of uncarboxylated forms of several VKDPs, such as matrix Gla protein (MGP), osteocalcin and PIVKA-II (protein induced by vitamin K absence or antagonism–II). Using these methods, several studies over the last two decades are unanimous in showing sub-clinical levels of vitamin K in CKD patients and its association with VC and CVD outcomes, with higher impact in hemodialysis patients [59–65] (further detailed below).

### Phosphate binders (PB) to control serum phosphate levels: high promise and low effectiveness

While many different classes of phosphate binders (PBs) are available and shown to be able to reduce serum P levels, a considerable uncertainty about the benefits and harms of specific PBs still remains [66]. In fact, inconsistent data is found in the literature, with some studies pointing for a decreased risk of all-cause mortality in patients treated with some PBs, particularly sevelamer, when compared to calcium-based phosphate binders (CBPBs) [67,68]. However, recent meta-analysis in adults with CKD, including dialysis and non-dialysis patients, revealed disappointing results for several PBs in terms of clinical outcomes, including cardiovascular death, myocardial infarction, stroke, fracture or coronary artery calcification [69,70]. Overall, there was no evidence of any PB lowering mortality or CVD events compared to placebo. Importantly, CBPBs such as calcium carbonate or calcium acetate, have been the first choice in therapeutic use, with a dual goal of fighting hyperphosphatemia and raising serum Ca levels to suppress PTH. However, there is increasing evidence that the use of CBPBs contributes to positive Ca balance and hypercalcemia promoting the progression of VC. Indeed, this is not unexpected since by binding P and decreasing serum P, Ca in CBPBs will also promote the synthesis of calcium-phosphate mineral, CaP. Also, although most P binding occurs in the gastrointestinal tract, some Ca is absorbed contributing to increase serum Ca levels and the formation of CaP in circulation. Several studies have shown that, when compared with non-CBPBs such as sevelamer and lanthanum carbonate, the use of CBPBs contributes to progressive coronary artery and aorta calcification [71–75]. Based on these evidences that CBPBs produce a positive calcium balance, recommendations from the Kidney Disease Outcomes Quality Initiative Guidelines (KDIGO) indicate that doses should not exceed 1500 mg/day of elemental calcium [76].

## Role of phosphate, calcium and calcium-phosphate mineral in vascular calcification: Who is the real culprit?

The notion that controlling hyperphosphatemia has limited results in terms of ameliorating VC, challenges the concept that P, per se, is the real culprit of VC in CKD patients. The biological rationale for the association between abnormal P homeostasis and CKD is based on the role of P as a primary stimulus for the transformation of VSMCs from the contractile to an osteochondrogenic phenotype, with calcifying capacity [77]. Also, increased apoptosis, generation of reactive oxygen species and impaired production of nitric oxide are linked to hyperphosphatemia, contributing to endothelial dysfunction [78]. However, some studies have shown that exogenous P alone is insufficient to induce VSMCs mineralization, others have reported increased mineralization in cultures under increased Ca levels and normal P conditions [79,80]. Several reports also describe a synergistic action of both Ca and P in accelerated and increased mineralization *in vitro* [80,81]. Explanations to conciliate these *in vitro* data are based on the formation of CaP crystals in culture conditions, which have been shown to induce cell differentiation and vascular mineralization [82,83]. Treatments with pyrophosphate or analogues, inhibit hydroxyapatite and nanocrystals formation and completely abrogate VC [80,82]. Clinical studies have in fact shown that the simultaneous control of Ca, P and PTH is crucial to decrease the mortality risk and cardiovascular hospitalization in dialysis patients [84,85].

While many studies have demonstrated the cell toxicity of CaP crystals, it became very clear that highly complex mechanisms exist to control the formation, maturation and pathogenicity of these mineral nucleation sites. This is in line with the currently accepted notion that VC is an active, naturally occurring, and tightly regulated multifactorial process, that must be actively inhibited (extensively reviewed elsewhere [16,86,87]). In fact, CaP nanocrystals are constituents of calciprotein particles (CPPs), mineralization-competent extracellular vesicles (EVs), and mineralized material deposited in the ECM of blood vessels. Furthermore, CaP crystals have been shown not only to induce VSMCs osteochondrogenic differentiation, but to promote pro-inflammatory reactions which increased pro-calcific responses, in a cycle were increased mineralization triggers inflammation and vice-versa. It is currently accepted that VC and inflammation not only play a key role in CKD pathophysiology and cardiovascular disease, but are also involved in a complex bidirectional crosstalk ultimately leading to disease progression. This clearly elevates the concept that CaP crystals inhibition is of vital importance in an overall context of VC management and cardiovascular clinical outcomes.

## Inhibition of calcium-phosphate mineral: the gold standard to manage vascular calcification in CKD?

The contribution of impaired bone metabolism and the precise mechanism responsible for VC in CKD have not been fully elucidated. Nevertheless, it is currently accepted that this is an active multifactorial process in which CaP mineral, mostly in the form of hydroxyapatite, is deposited in the ECM of the vascular tree, resembling bone formation. Under physiological conditions, local and systemic inhibitors of mineral formation act to prevent widespread tissue calcification by influencing VSMCs osteochondrogenic differentiation, formation of calcifying competent EVs, maturation of calciprotein particles (CPPs) and ECM crystal growth. Despite the numerous molecules already identified with a calcification inhibitory function, here we will highlight the role of matrix gla protein (MGP), fetuin-A (or alpha 2-Heremans-Schmid glycoprotein, AHSG), and Gla-rich protein (GRP), also known as upper zone of growth plate and cartilage matrix associated protein (UCMA) [88,89]. In fact, recent evidences point to an interconnected action of these proteins in several calcifying driver events. In addition, insufficient vitamin K levels in CKD patients are correlated with decreased functionality of MGP and GRP. Although the mechanisms underlying MGP, GRP and fetuin-A calcification inhibitory function might have distinct molecular pathways, they are all involved in the inhibition of CaP mineral growth. Also, they can be associated with VSMCs-released EVs and CPPs, and VSMCs osteochondrogenic differentiation.

### Role of calcification inhibitors and vitamin K in vascular calcification

MGP and GRP are vitamin K-dependent protein (VKDP) synthetized by VSMCs in vascular tissues. Fetuin-A is a liver-derived blood cysteine protease inhibitor uptake from circulation by VSMCs. MGP and fetuin-A are longstanding recognized vascular calcification inhibitors [90,91]. GRP was more recently shown to function both as a calcification inhibitor and an anti-inflammatory agent in the cardiovascular and articular systems [92–94]. Functional *in vivo* and *in vitro* models have established the importance of these inhibitors in vascular calcification, with a preponderant role at tissue and systemic levels. Knockout mice for MGP (MGP-/-) result in massive vascular calcification affecting the main arteries and death within 8 weeks of birth [95]. Restoration of MGP expression in VSMCs from MGP-/- rescued the arterial calcification phenotype [96]. Fetuin-A deficient mice combined with a calcification-sensitive mouse strain or a mineral and vitamin D rich diet, results in progressive and lethal calcification of soft tissues, including kidneys, skin, heart and vasculature [97]. The role of GRP as inferred by animal models is still being explored. Similarly to fetuin-A, GRP knockout mice (GRP-/-) without additional challenging conditions such as aging or disease, present a normal phenotype in terms of skeletal development [98]. However, after destabilization of the medial meniscus, GRP-/- mice develop a severe osteoarthritis phenotype clearly indicating a chondroprotective effect for GRP [99]. Also, VSMCs from GRP-/- mice exposed to calcifying conditions show increased mineralization and expression of osteochondrogenic markers [100]. In addition, using a human *ex vivo* model of VC, γ-carboxylated GRP was shown to inhibit calcification and osteochondrogenic differentiation [92]. These studies confirm a preponderant role for GRP as an inhibitor of VC. This is also in line with functional studies in zebrafish suggesting an essential role of GRP in skeletal development and calciﬁcation [101]. It should be noted that the use of animal models has several shortcomings in direct translation to the human situation. In the case of GRP, several differences in terms of protein and gene expression patterns and different isoforms in mice and human, might imply different functional mechanisms [88,102,103]. Of note, despite the impressive phenotype of MGP-/- mice, loss-of-function mutations in the human MGP gene, known as the Keutel syndrome, results in non-lethal abnormal soft tissue calcifications [104], suggesting that additional or compensatory mechanisms of pathological mineralization inhibition might exist in human.

Most of the inhibitory function, common to these three proteins, has been attributed to a direct interaction with the mineral phase and the prevention of crystal maturation and precipitation. Fetuin-A binds small clusters of Ca and P in blood, forming soluble protein mineral particles, CPPs [also known as fetuin-mineral complex (FMC)], preventing mineral growth, aggregation and precipitation [91]. MGP and GRP directly interact with calcium crystals inhibiting their growth and maturation through its calcium-binding Gla residues [105–108]. This is the reason why the activity of VKDPs is well known to depend on their γ-carboxylation status, and non- or undercarboxylated protein forms are often regarded as non-functional, accumulating at sites of pathological calcification. Of note, while human MGP contains 5 possible Gla residues, human GRP contains 15 putative Gla residues, implying different calcium-binding properties [109,110]. While the association between MGP carboxylation and vascular calcification has been extensively studied [58,90,111], undercarboxylation of GRP was more recently associated with several calcification-related diseases, as calcific aortic valve disease [92], osteoarthritis [93,102] and certain cancers [107]. Importantly, *in vitro* studies have shown that only γ-carboxylated GRP display anti-mineralization capacity [92,93]. This is in line with the widely demonstrated role of vitamin K in cardiovascular calcification, which has been consistently associated to its function as co-factor for γ-carboxylation reaction of VKDPs. Low concentrations of vitamin K in CKD patients have been associated with (i) dietary restrictions; (ii) storage exhaustion due to a high demand on VKDP activity involved in the regulation of VC related processes; (iii) the use of anticoagulant therapy with vitamin K antagonists such as warfarin; (iv) the use of phosphate binders that can also bind vitamin K inducing vitamin K-deficiency [112–116]. The overall poor vitamin K status has been widely correlated with the uncarboxylation of target calcification inhibitors such as MGP, and consequent increased VC and poor cardiovascular prognostic. In this line, supplementation with vitamin K has been proposed as a complementary nutrient to improve vascular health in the general population and in CKD patients. In the population-based Rotterdam study, vitamin K2 intake was found inversely related to all-cause mortality and severe aortic calcification [117]. Also, vitamin K treatment was shown to inhibit warfarin-induced VC [118–120]. In CKD stage 3-5 and in hemodialysis patients, vitamin K2 intake has been shown to dose-dependently reduce circulating levels of uncarboxylated MGP [121,122]. Novel clinical trials are underway to evaluate the effect of vitamin K1 administration in hemodialysis patients in relation to the progress of coronary and aortal calcification (VitaVasK and iPACKHD).

### Calcium-phosphate mineral in extracellular vesicles and calciprotein particles

The earliest phase of VC has been shown to occur via the secretion of calcifying-competent EVs, which nucleate calcium phosphate crystals with consequent propagation of calcification in the ECM [123,124]. While in calcified arteries mineral-containing EVs localize in close proximity to elastin and collagen fibrils [125,126], in healthy arteries EVs released by VSMCs are devoid of mineral. It has been suggested that under normal conditions, VSMCs-derived EVs do not calcify due to their loading with mineralization inhibitors. Calcifying EVs released from VSMCs with increased calcium loading are known to have decreased levels of MGP, fetuin-A and GRP, resulting in a deficient inhibition capacity of CaP crystals nucleation and growth [125,92]. More recently, decreased levels of GRP and fetuin-A in circulating EVs isolated from CKD stage 5 patients were associated with increased calcification of VSMCs [108]. Furthermore, removal of EVs from healthy serum clearly promoted VC, suggesting that these circulating nanoparticles containing higher levels of GRP and Fetuin-A constitute a powerful anti-mineralization system that is able to regulate mineral formation both at systemic and tissue levels [108].

In fact, VC at tissue level is highly influenced by systemic factors. Serum is supersaturated with Ca and P, and the discovery of CPPs in circulation- predominantly composed of fetuin-A, minerals and calcium-regulatory proteins, initially reported in rats [127,128] and further identified in CKD patients [129], highlighted a mechanism by which extraskeletal mineralization is prevented. These entities are considered mineral chaperones with a role in the stabilization, transport and recycling of insoluble CaP mineral in blood, preventing growth, aggregation and precipitation of the mineral crystal. In addition to fetuin-A, which has been extensively shown to have a preponderant role in CPPs formation [130,131], MGP and GRP are also constitutive components of CPPs [108,132]. Several *in vitro* studies have shown that CPP formation is biphasic and two types of CPPs, termed primary or CPP-I, and secondary or CPP-II, can be identified with different size, composition and morphology [130,132]. CPP-I start with the formation of calciprotein monomers, constituted by aggregated small clusters of fetuin-A-bound mineral ions, consisting on spherical nanoparticles containing amorphous calcium phosphate. These CPP-I nanoparticles can undergo transformation into CPP-II which have a more densely needle-like shape and contain crystalline mineral. It has been suggested that in healthy individuals CPP-I may form spontaneously in all tissues, transported in the blood and removed by class A scavenger receptor–mediated pathways, or locally used by osteoblasts during bone formation [133]. In cases of chronic dysregulation of mineral metabolism such as in CKD, increased transformation and accumulation of CPP-II have been linked to toxicity with consequent increase in vascular calcification and inflammation. Several tests have been developed to indirectly determine CPP levels in serum, either through the fetuin-A reduction ratio or through the serum calcification propensity (T_50_) [129,134]. Also, the measurement of CPP in blood has been proposed as a prognostic marker in CKD as predictive of mortality [135]. CPPs can be detected since early stages of CKD when baseline serum phosphate is still within the normal range, and increasing with worsening renal function [129,135,136]. Moreover, correlations between circulating CPP levels and coronary artery calciﬁcation scores, aortic pulse wave velocity, aortic stiffness and serum markers for inflammation have been described [129,135–137]. In patients with end-stage renal disease, the reduction of PTH also results in a reduction in serum CPP [129]. Possible explanations for the relation between CPP levels and VC have been suggested. These are focused either on the decreased levels of free circulating fetuin-A due to increased CPP levels, that otherwise would prevent VC at the vascular wall, or on the toxicity of CPPs. In fact, several lines of evidence point for a pathogenic role of CPP-II containing a more crystalline and insoluble hydroxyapatite-like mineral phase, where circulating mineralization inhibitors play a crucial role to block mineral nucleation, growth and maturation. Synthetic CPP-II, and not CPP-I, have been shown to induce VSMCs calcification, and serum derived CPPs have a higher protective effect compared to synthetic CPPs in macrophage activation [138–140]. Recently, CPP particles from healthy individuals were shown to resemble CPP-I, while those from CKD patients had CPP-II-like features with increased mineral maturation and decreased levels of fetuin-A and GRP [108]. These CKD-derived CPP-II particles are uptake by VSMCs and promote calcification by inducing osteochondrogenic differentiation and inflammation processes. Importantly, incubation of CKD-CPPs with γ-carboxylated GRP rescued the calcification, osteogenic differentiation and inflammatory status induced in VSMCs [108].

### A common anti-mineralization system acting locally and systemically?

Overall, fetuin-A, GRP and MGP are all present in EVs, in CPPs and in the calcified ECM of blood vessels, sharing functional mechanisms and undoubtedly associated with the inhibition of VC. Furthermore, several evidences suggest that these three proteins are constituents of a powerful anti-mineralization system with synergistic effects to regulate the dynamics of mineral formation, both at systemic and tissue levels, to prevent ectopic mineral deposition. Co-immunoprecipitation assays have demonstrated the presence of a protein complex containing GRP, fetuin-A, and MGP at sites of aortic valve calcification, but also in non-calcified aortas and in EVs isolated from VSMCs cultured in control conditions, indicating a constitutive physiological function [92,108]. *In vitro* assays have shown that mineral crystal formation and maturation is dependent on the simultaneous presence of γ-carboxylated GRP, fetuin-A and MGP [108]. It is also noteworthy the similarities on the inhibitory mechanism of VSMCs osteochondrogenic differentiation. Fetuin-A, MGP, and more recently GRP, were shown to inhibit osteochondrogenic differentiation of VSMCs via bone morphogenetic proteins (BMP)-dependent signaling, with direct binding to the potent osteogenic differentiation factor BMP2 [100,141–144]. However, it remains to be clarified whether fetuin-A, GRP and MGP act as a single protein complex on BMP2 inhibition. Collectively, these data support the theory that the role of mineralization inhibitors to block mineral nucleation is crucial to inhibit VC, possibly through a common mechanism acting at systemic and tissue levels. Disturbances on the levels or functionality of these proteins are associated with increased VC. Fetuin-A deﬁciency has been consistently associated with increased arterial calciﬁcation scores and higher mortality rates, and in CKD, low circulating fetuin-A levels are associated with progressive aortic stiffening and calciﬁcation [145–148]. The inactive form of MGP, dephosphorylated and uncarboxylated (dp-ucMGP), has been correlated with CKD severity and positively associated with VC [121,149–151]. A recent observational study showed that total GRP serum levels correlate with the deterioration of renal function, and was an independent risk factor of VC in diabetic patients with low to moderate CKD [152].

## The crosstalk between vascular calcification and inflammation: a plausible explanation for the burden of CVD in CKD

In addition to the complexity of mechanisms involved on VC initiation and progression, it is currently accepted that it cannot be regarded as an isolated pathological process, with several studies providing compelling evidence that VC is highly interconnected with inflammation. In fact, it has been suggested that pathological calcification and chronic inflammation are involved in a positive feed-back loop driving disease progression [153–155].

**Figure 3:**
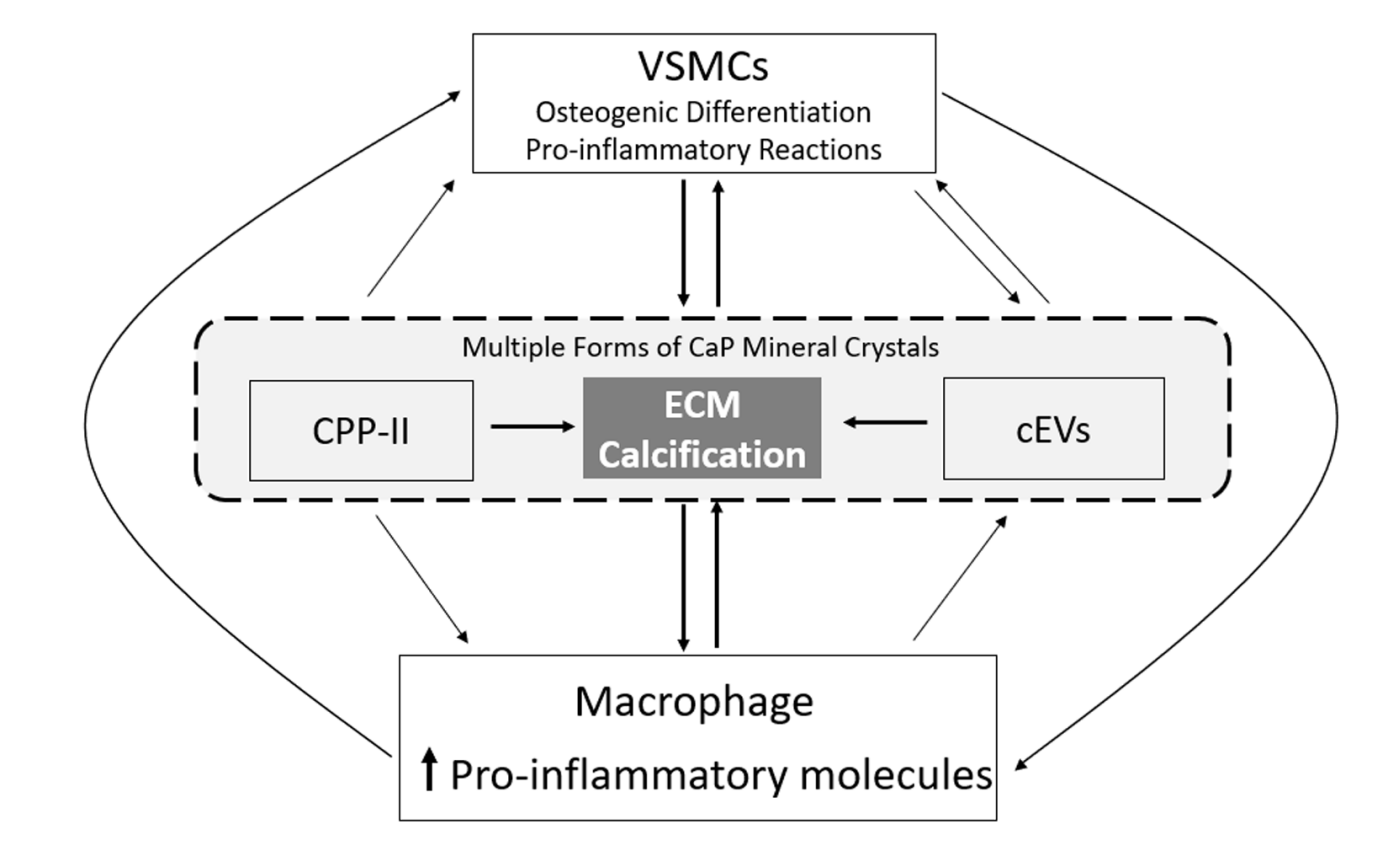
**The vascular calcification-inflammation cycle.** Calcium-phosphate (CaP) mineral is present in secondary calciprotein particles (CPP-II), in calcifying extracellular vesicles (cEVs) and in the extracellular matrix (ECM) of blood vessels. All these forms of CaP mineral are able to induce pro-inflammatory responses in immune and VSMs cells, and the osteogenic differentiation of VSMCs. In turn, macrophage pro-inflammatory responses contribute to increased vascular calcification through the release of cEVs and inducing osteogenic differentiation of VSMCs, while osteogenic VSMCs drive ECM calcification through the release of cEVs and increase in macrophage pro-inflammatory responses, in a vicious cycle.

In CKD, persistent microinflammation has been recognized as an important pathophysiological component, contributing for cardiovascular disease and mortality. Increased C-reactive protein (CRP), which is a predictor of cardiovascular risk factors and death in the general population, has been long associated with increased vascular calcification and mortality in hemodialysis patients [156–159]. Inverse correlations between GFR and levels of proinflammatory cytokines, such as IL-1β, IL-6 and TNF-α, clearly demonstrate the role of inflammation on CKD development [160]. In a cohort of hemodialysis patients, a pattern of high proinflammatory cytokines IL-1β, IL-6, and TNF-α, in combination with low anti-inflammatory parameters including IL-2, IL-4, IL-5, IL-12, were associated with decreased survival [161]. In fact, several of these inflammation-related biomolecules have been proposed as biomarkers for cardiovascular mortality in CKD, although differing in their predictive value [162,163]. The high levels of inflammatory molecules in CKD might be explained, at least in part, with increased production at tissue level such as at the vascular wall. In atherosclerotic lesions, the accumulation of macrophages within the vascular wall has been consistently co-localized with calcium deposits and associated with various phases of calcification [164,165]. More pronounced macrophage infiltration and higher CRP were described in coronary artery lesions of CKD [166]. Early stages of CKD are already associated with up-regulation of proinflammatory and pro-osteogenic molecules in the vascular wall and calcification of the aortic media [167]. In fact, several lines of evidence indicate that inflammation triggers and precedes osteogenic conversion of VSMCs and the release of calcifying EVs, promoting the calcification process. It is likely that the effect of inflammation on VC occurs at multiple and interconnected levels. It has been proposed that inflammation might regulate VC, at least in part, through activation of an endoplasmic reticulum stress pathway, which in turn may increase inorganic phosphate uptake, leading to increased VSMCs osteogenic differentiation and increased mineral deposition [168]. Activated macrophages at sites of tissue damage also produce high levels of matrix metalloproteinases, cysteine endoproteases and cytokines, which will enhance elastin and collagen degradation. These processes leading to remodelling and structural changes of the ECM, will contribute to create a nidus for CaP crystal growth [169–171]. Also, macrophages have been shown to regulate VC through the release of osteogenic factors capable of inducing VSMCs osteogenic differentiation [172,173]. Among the many soluble factors released by activated macrophages and known to be elevated in CKD, TNFα and also IL-1β are reported to enhance VSMCs osteogenic activity by increasing BMP2 production [173]. In a scenario of reduced MGP and GRP levels (or functionality) to inhibit BMP2 osteogenic signaling, VSMCs osteogenic differentiation is potentiated, and further aggravated through the release of calcifying competent EVs lacking MGP and GRP inhibitors. Also interesting is the possible direct role of macrophages on vascular calcification, through the release of calcifying EVs containing hydroxyapatite nucleation sites, and capable of mineralization [174]. These macrophage- released EVs are loaded with mineralization related factors, and have an increased Ca content and alkaline phosphatase activity. Interestingly, GRP was shown to be present, at protein and mRNA levels, in EVs released by THP-1 differentiated macrophages [94]. Although additional studies are required to understand the role of GRP in macrophage-derived EVs with calcifying capacity, it is licit to speculate that GRP might also be involved on the inhibition of mineral nucleation. In fact, GRP was shown to be synthetized and γ-carboxylated in the majority of human immune system cells, including monocytes and macrophages [94], and the anti-inflammatory properties of GRP have been highlighted. Treatments with exogenous GRP or GRP overexpression were both shown to be able to decrease the pro-inflammatory response of THP-1 monocytes/ macrophages and of articular cells, by down-regulating pro-inflammatory molecules such as TNFα, IL-1β and nuclear factor kappa B [93,94]. A similar down-regulation of mediators of inflammation and inflammatory cytokines was observed when basic calcium phosphate (BCP) crystals were coated with GRP, clearly indicating GRP as a crosstalk agent between inflammatory and calcifying processes [93,94]. In fact, while macrophages are key players signaling ECM degradation, calcification and cells differentiation, a multitude of CaP crystals have been shown to promote pro-inflammatory responses, which again will affect VC, in a calcification-inflammation pathological feeding cycle (Figure 3). Micro-calcifications have been proposed to be involved in macrophage recruitment in early stages of atherosclerosis development [164], and BCP crystals stimulate macrophages to produce pro-inflammatory cytokines affecting VSMCs differentiation [94,175,176]. BCP crystals were shown to directly interact with macrophages, inducing an increased production of TNFα, IL-1β and IL-8, which in turn stimulate the activation of endothelial cells and recruitment of mononuclear cells [175], amplifying the immune response. Coating of BCP particles with fetuin-A and GRP, resembling CPPs, was shown to decrease pro-inflammatory responses when compared to naked crystals [93,94,140], and synthetic secondary CPP particles were shown to induce an up-regulation of TNFα accompanied by increased calcification [138,139]. More recently, a link between inflammation and calcification in CKD was highlighted. CPPs isolated from CKD stage 5 patients, containing reduced levels of GRP and fetuin-A, were shown to be highly osteogenic driven factors, with the capacity to increase VSMCs calcification by promoting osteogenic differentiation and inflammation [108]. Importantly, the dual capacity of GRP to act as an anti-inflammatory agent and an inhibitor of vascular calcification, was clearly demonstrated when the calcification/osteogenic differentiation and inflammatory status induced in VSMCs was rescued by supplementation of those CKD5-CPPs with GRP [108]. While the role of fetuin-A in inflammation has also been demonstrated, functioning as an acute phase reactant protein (reviewed in [91,177]), the research data on the role of MGP in inflammation is still quite incipient. Only a few studies have reported a possible association between increased levels of MGP and reduced inflammation, by inhibiting BMP-induced inflammatory pathway [178,179]. More recently, results from a mouse experimental colitis model shown that MGP was implicated on the suppression of T cells proliferation and cytokine production in Crohn’s disease [180]. Also, the association between circulating levels of MGP and inflammatory markers is still quite limited and inconsistent [181–183]. While an inverse association between dp-ucMGP (reflecting vitamin K status) and inflammatory markers is shown in some observational studies [184], long term vitamin K2 supplementation do not show a correlation [185]. In fact, the association between vitamin K levels, either K1 or K2, and inflammation is still debatable [186,187].

In this context, it is also noteworthy the proposed function of vitamin K as an anti-inflammatory agent, as mediated by a direct vitamin K effect rather than through VKDPs functionalization [188–190]. In this regard it is interesting to note that the anti-inflammatory function of GRP seems to be independent of its γ-carboxylation status [93,94]. Considering the conflicting results and the reduced number of available randomized studies addressing causality between vitamin K status and inflammation [191–193], additional studies are required to establish the direct role of vitamin K in inflammation, particularly concerning the inflammation/vascular calcification cycle in CKD-CVD outcomes.

Remarkably, this inflammation/vascular calcification crosstalk described in CKD pathology, share many similarities with the aging process in the general population, including the inflammaging and VSMCs senescence [194,195]. Inflammaging is a recently adopted term do define a state of low grade chronic inflammatory condition, associated with a significant risk factor for morbidity and mortality in the elderly (reviewed in [196]). Cellular senescence, in general, has been proposed as a potential mechanism of aging and age-related diseases, which can be triggered by a number of mechanisms and leading to an altered secretome, termed the senescence-associated secretory phenotype (SASP) (reviewed in [195,197]). In the particular case of VSMCs, senescence has been shown to enhance vascular calcification and inflammation, with pro-calcific and pro-inflammatory SASPs [195,198,199]. These SASPs share substantial similarities with the osteochondrogenic phenotype of VSMCs under uremic conditions, including the overexpression of bone-related, inflammation and extracellular matrix degradation markers. Also, microvesicles isolated from elderly people and from senescent endothelial cells, characterized by high levels of calcification-related proteins and increased calcium content, were shown to promote calcification of VSMCs [200]. In fact, VSMCs senescence and associated SASP have been suggested to contribute for chronic vascular inﬂammation and calcification, loss of arterial function, and the development of age-related diseases [195–197]. Thus, it has been suggested that altered vascular health under CKD settings might represent an example of premature aging [194,195]. In this context, it could be conceivable that new knowledge about molecular mechanisms, such as the crosstalk between VC and inflammation, in CKD, might shed new horizons on the aging process, and vice-versa. It is clear that several biomolecules, including mineralization inhibitors such as GRP, act in a highly complex and coordinated network between inflammation and calcification processes, and that a deeper knowledge of this complex crosstalk is crucial to the development of new therapies and biomarkers that will benefit the populations at high risk for cardiovascular diseases.

## Conclusion and perspectives

Cardiovascular diseases continue to be the leading cause of death in all CKD stages, despite all research efforts to improve our knowledge on the molecular mechanisms and processes involved on its development and progression. Traditional risk factors are widely accepted as insufficient to predict and prevent CVD events in CKD, and great attention is now focused on the benefits of additional nontraditional risk factors, such as VC and inflammation, which are considered potentially valuable prognostic tools and targets for therapeutics and management of cardiovascular risk. Over the years, and accompanying the advances on basic science, several therapeutic approaches have been proposed as the holy grail for cardiovascular diseases in CKD. Some of these have focused on the control of P through diet and/or phosphate binders, others have hyperparathyroidism as the main therapeutic target, either through increase serum Ca with CPBDs or with activated vitamin D (calcitrol). Also, secondary prevention interventions aiming to reduce the risk of cardiovascular events in CKD, have been focused on controlling hypertension and dyslipidemia. Angiotensin-converting enzyme inhibitors (ACEis) and angiotensin receptor blockers (ARBs) have been used as a first therapeutic line in hypertension due to its well-known renoprotective effect, and still comprise the standard of care for nephropathy treatment. Statins, well known inhibitors of cholesterol endogenous production, are currently used as lipid controlling therapies in patients with mild to moderate CKD. Unfortunately, several drawbacks have been identified in all these therapeutic approaches proposed to manage cardiovascular diseases in CKD, with very limited effective results. In addition to what was already discussed, treatment with moderate- to high-dose calcitriol might led to hypercalcemia and hyperphosphatemia [201–203], while treatment with multiple antihypertensive agents fail to reach target blood pressure in some CKD patients [204]. Also, lipids control has not been as successful approach as predicted, with failure to reduce the cardiovascular morbidity and mortality in the end stage renal disease population [205,206].

We propose that incorporating multiple non-traditional risk factors, targeting simultaneously different processes involved in CVD development and progression such as vascular calcification and inflammation, would pave the way to i) develop new biomarkers to identify patients with increased cardiovascular risk, ii) improve traditional multivariate risk assessment, and iii) establish new effective and safe therapies. While the complexity between vascular calcification and inflammation poses huge scientific challenges to decipher crosstalk signals and interconnected molecular pathways, it comprises exciting expectations for the development of new biomarkers, effective early therapeutic and preventive intervention measures, to manage cardiovascular diseases in CKD.
